# *De novo* biosynthesis of 1,5-diamino-2-hydroxypentane from glucose via combination of fermentation and whole-cell catalysis

**DOI:** 10.1016/j.synbio.2026.01.036

**Published:** 2026-02-14

**Authors:** Zhijie Zheng, Zhongliang Chen, Tongqing Tang, Haoyu Lu, Qian Xu, Zixun Yang, Wei Liu, Chaoqiang Wu, Shewei Hu, Feifei Chen, Alei Zhang, Kequan Chen

**Affiliations:** a State Key Laboratory of Materials-Oriented Chemical Engineering, Nanjing Tech University, Nanjing, 211816, China; b College of Biotechnology and Pharmaceutical Engineering, Nanjing Tech University, Nanjing, 211816, China; c Department of Biology and Food Engineering, Bozhou University, Bozhou, 236800, China

**Keywords:** 1,5-Diamino-2-hydroxypentane, 3-Hydroxylysine, l-lysine 3-hydroxylase, SpLDC, Pathway enhancement

## Abstract

1,5-Diamino-2-hydroxypentane (2-OH-PDA) is a novel aliphatic β-amino alcohol with potential for a wide range of applications, and its efficient and green synthesis has attracted increasing attention. In this study, we developed a biosynthetic pathway for 2-OH-PDA from glucose. Firstly, the Fe^2+^/α-ketoglutarate (α-KG)-dependent l-Lysine-3-hydroxylase (K3H) was heterologously expressed in *E coli* NT1003 capable of highly producing l-lysine to construct a biosynthetic pathway strain of 3-hydroxylysine (3-OH-lysine). By intensifying the pathway, introducing a glutamate oxidation system, and optimizing the fermentation conditions, 40.2 g/L 3-OH-lysine were achieved in the fed-batch fermentation. Then, lysine decarboxylase (SpLDC) was used to catalyze the decarboxylation of 3-OH-lysine to 2-OH-PDA, yielding 29.2 g/L with a molar conversion efficiency of 99.7%. This study provides an economical, efficient and green route for the biosynthesis of 2-OH-PDA.

## Graphical abstract

**Figure:**
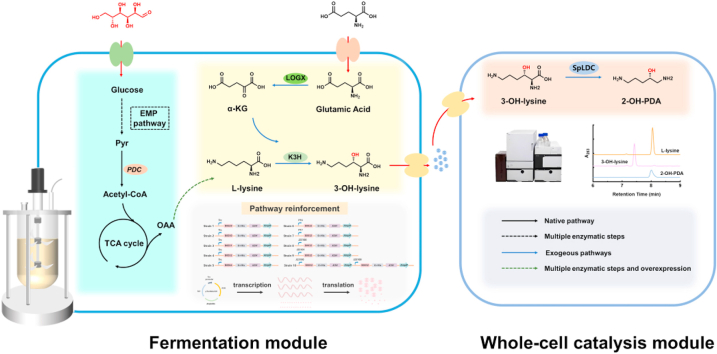

## 1 Introduction

β-amino alcohols, featuring both amino (–NH_2_) and hydroxyl (–OH) functionalities on adjacent carbon atoms, are versatile building blocks in synthetic and medicinal chemistry [[1], [2], [3], [4], [5]]. Their dual hydrogen-bonding capacity and metal-coordinating properties make them indispensable structural motifs in a wide range of bioactive molecules [6]. Notably, the β-amino alcohol scaffold serves as a key pharmacophore in several clinically important antibiotics, such as chloramphenicol and thiamphenicol [7]. Beyond their role as pharmacophores, β-amino alcohols also serve as crucial intermediates in pharmaceutical synthesis [[8], [9], [10]]. A representative example is *(S)*-2-amino-3-methyl-1-butanol, which functions as a chiral precursor in the industrial production of Elvitegravir, an FDA-approved HIV-1 integrase inhibitor [3,11].

Although aromatic and cyclic β-amino alcohols have been extensively studied, aliphatic β-amino alcohols with novel architectures and multiple functional groups remain scarcely reported and are far from being fully exploited [12,13]. 1,5-diamino-2-hydroxypentane (2-OH-PDA) is a target molecule based on its unique molecular structure compared to conventional biobased diamines like putrescine or cadaverine. 2-OH-PDA features a distinctive arrangement comprising a β-amino alcohol moiety and a primary amine group spatially separated within a single aliphatic chain. This structural dissimilarity grants 2-OH-PDA distinct reactivity, enabling its involvement in both hydroxyl- and amine-mediated chemical properties such as heightened polarity, metal-complexing capabilities, and adjustable cross-linking, which has potential applications in asymmetric synthesis, intelligent polymer materials, and eco-friendly curing formulations [5]. Its chemical synthesis results in poor regioselectivity, limited stereoselectivity, demanding reaction conditions, and unstable reaction intermediates. Thus, no efficient chemical synthesis of 2-OH-PDA has been reported to date. To overcome these obstacles, the exploration of biosynethetic routes is imperative.

Baud et al. reported an *in vitro* enzymatic cascade using purified lysine hydroxylase (KDO1) and pyridoxal phosphate-dependent lysine decarboxylase (LDC) to directly convert l-lysine into 2-OH-PDA, achieving a high yield of 93% [14]. However, the requirement for purified enzymes entails high costs, limiting its feasibility for large-scale industrial application [15]. To address this challenge, previous study developed an engineered *Escherichia coli* whole-cell cascade system that integrates K3H-mediated lysine hydroxylation and CadA-mediated decarboxylation, achieving a 2-OH-PDA titer of 80.5 g/L from l-lysine of 159.6 g/L and α-KG of 191.5 g/L with a molar yield of 62.6% toward l-lysine [2]. Despite the impressive yields, the exogenously supplemented with two high-cost substrates (l-lysine >10 USD/kg, α-KG > 50 USD/kg) and complex process still limit the economic viability of the method.

In this study, we establish the first *de novo* two-step biosynthetic pathway for 2-OH-PDA directly from glucose (∼0.4–0.8 USD/kg) and l-glutamate (∼2–3 USD/kg), thereby eliminating the dependence on costly exogenous l-lysine and α-KG. Firstly, an l-lysine-producing strain, *E. coli* NT1003, was used as a chassis for K3H expression [16]. Subsequent systematic optimization of genetic elements (including promoter, ribosome binding site (RBS), and gene copy number) and fermentation parameters was investigated for enhancing 3-hydroxylysine (3-OH-lysine) yield. Furthermore, to enable cost-effective α-KG supply, l-glutamate oxidase and catalase were co-expressed to convert inexpensive exogenous l-glutamate into α-KG and eliminate H_2_O_2_ produced during the oxidase reaction [17,18]. Finally, whole-cell biocatalysts expressing lysine decarboxylase were employed to convert 3-OH-lysine into 2-OH-PDA. This study provids a scalable and economically viable route for the high-value scaffold.

## 2 Materials and methods

### 2.1 Chemicals

l-lysine hydrochloride, α-ketoglutaric acid (α-KG), (NH_4_)_2_Fe(SO_4_)_2_·6H_2_O, glutamic acid and sodium ascorbate were purchased from Sigma-Aldrich Inc. (Shanghai, China). All other chemicals and reagents were purchased from local suppliers (Nanjing, China) and were analytical grade.

### 2.2 Strains, plasmids and primers

*Escherichia coli* DH5α was employed as the cloning host for plasmid construction. *E*. *coli* BL21 (DE3) was used for protein expression. l-lysine-producing strain *E*. *coli* NT1003 was provided by Professor Yu Long and used as chassis for 3-OH-lysine biosynthesis via K3H expression [16].

Plasmids utilized in this study, including all recombinant constructs, are listed in Table S1. Corresponding primers are detailed in Table S2.

### 2.3 Construction of the strain for 3-OH-lysine production

The *K3H* gene from *Kocuria radiotolerans* was codon-optimized for expression in *Escherichia coli* and synthesized by Anhui General Biotech Co., Ltd. The gene was initially cloned into the pRSFDuet-1 vector between the *Nde*I and *Xho*I sites. Subsequently, the *K3H* fragment was amplified by PCR and inserted into the pTrc99A vector between the *Eco*RI and *Hin*dIII sites via homologous recombination, yielding the recombinant plasmid pTrc99A-*K3H*. This recombinant plasmid was transformed into the *E. coli* NT1003 strain, resulting in *E. coli* NT1003-pTrc99A-*K3H*.

### 2.4 Fermentation optimization of 3-OH-lysine production in shake-flask

A single colony of the recombinant strain *E. coli* NT1003-pTrc99A-*K3H* was inoculated into 5 mL of LB liquid medium and cultured at 37 °C with 200 rpm for 12 h. Subsequently, 1 mL of the seed culture was transferred to a 500 mL flask containing 100 mL of fresh LB medium and incubated at 37 °C and 200 rpm. When the optical density at 600 nm (OD_600_) reached 0.6–0.8, isopropyl-β-d-thiogalactopyranoside (IPTG) was added to a final concentration of 0.5 mM, followed by further induction at 25 °C for 24 h.

To investigate the effect of key parameters on 3-OH-lysine production, experiments were conducted in 500 mL shake flasks by varying only one factor while keeping others constant. The conditions tested included induction temperature (15, 20, 25, 30, 35, and 40 °C), fermentation culture duration (12, 24, 36, 48, 60, and 72 h), IPTG concentration (0, 0.1, 0.2, 0.4, 0.6, 0.8, and 1.0 mM), initial glucose concentration (5, 10, 15, 20, and 25 g/L), α-KG concentration (0, 50, 100, 150, and 200 mM), and Fe^2+^ concentration (0, 0.5, 1, 2, 5, and 10 mM). All experiments were performed in triplicate.

### 2.5 Genetic engineering enhanced 3-OH-lysine biosynthesis

The expression of *K3H* gene at the genetic level was optimized. Firstly, to assess translational efficiency effects, four additional RBS sequences (B0029, B0030, B0032, and B0064) with varying strengths were selected from the iGEM Registry (http://parts.igem.org/Ribosome_Binding_Sites/Catalog). Using pTrc99A-*K3H* (containing the native B0031 RBS) as a template, inverse PCR was performed with primers designed using SnapGene software to replace the original RBS. Each resulting plasmid construct was transformed into *E. coli* NT1003 to generate mutant strains for subsequent analysis.

Subsequently, the influence of promoter strength on transcriptional activity was investigated. Four promoters (PJ23100, PJ23101, P16, and P17) with different characterized intensities were selected. Using the plasmid carrying the most effective RBS variant as template, inverse PCR was again employed to replace the native trc promoter. Each promoter-recombinant plasmid was transformed into *E. coli* NT1003 to create a series of strains for further evaluation.

Finally, to investigate the effects of increased gene dosage, a dual-copy *K3H* expression cassette was constructed. Using the parental plasmid containing the optimal promoter-RBS combination as a template, reverse PCR was performed with primers designed to introduce tandem repeats of the expression unit. The resulting linear product was self-ligated via in vivo homologous recombination following transformation into *E. coli* NT1003, yielding a plasmid carrying two consecutive copies of the *K3H* expression cassette.

### 2.6 Construction of an l-glutamate-driven α-KG supply system

To enable cost-effective supply of α-KG, an enzymatic regeneration system was constructed by co-expressing l-glutamate oxidase (LGOX) from *Streptomyces viridochromogenes* and catalase (CAT) from *Bacillus pumilus*. The native T7 promoters were replaced with the trc promoter by reverse PCR to allow constitutive expression in non-T7 systems. The recombinant plasmid, pRSFDuet-trc-*LGOX*-*CAT*, was verified by sequencing.

The plasmid was co-transformed with an engineered K3H expression construct optimized for promoter, RBS, and gene copy number into *E*. *coli* NT1003 to generate a dual-plasmid strain. The functionality of the α-KG supply system was evaluated at the shake flask by comparing 3-OH-lysine yields between this strain and control strain harboring only the K3H expression plasmid. All cultures were maintained under identical conditions, with 25 mM l-glutamic acid added at induction and replenished every 12 h.

### 2.7 Fermentation of 3-OH-lysine in a 5-L bioreactor

To evaluate the performance of the integrated α-KG supply and lysine hydroxylation pathways under controlled bioreactor conditions, fed-batch fermentation was scaled up to a 5-L bioreactor.

Seeds were prepared in 100 mL of LB medium in 500 mL shake flasks at 37 °C and 200 rpm for 12 h, then transferred to a 5 L fermenter containing 2.5 L of defined fermentation medium (20 g/L glucose, 10 g/L (NH_4_)_2_SO_4_, 5 g/L peptone, 2 g/L yeast extract, 0.5 g/L KCl, 0.032 g/L FeSO_4_·7H_2_O, 1.6 g/L MgSO_4_·7H_2_O, 0.086 g/L ZnSO_4_·7H_2_O, 0.077 g/L CuSO_4_, 0.032 g/L MnSO_4_·H_2_O, 10 g/L CaCO_3_, 0.3 g/L methionine, 0.1 g/L threonine, 0.06 g/L vitamin B_1_, 0.01 g/L niacinamide, 30 μg/L biotin).

The initial fermentation temperature was 37 °C. The pH was maintained at 7.0 by automatic addition of 25% (w/v) NH_4_OH. Dissolved oxygen (DO) was kept above 30% by adjusting the agitation speed (300–800 rpm) and aeration rate (1.0–4.0 vvm). The fed-batch was initiated when residual glucose was exhausted (8–10 h). A feed solution containing 500 g/L glucose and 250 g/L (NH_4_)_2_SO_4_ was continuously supplied, and the feed rate was adjusted to maintain glucose concentration below 5 g/L. When the OD_600_ reached approximately 10, 0.5 mM IPTG was added to induce protein expression. Simultaneously, 25 mM l-glutamate was supplemented based on preliminary shake-flask experiments (25 mM l-glutamate replenished every 12 h to ensure sufficient α-KG supply), and the temperature was shifted to 25 °C. Feeding was terminated when 3-OH-lysine concentration increased slowly, and the culture was then cultured for an additional 6 h to allow conversion of residual substrates. Finally, the fermentation broth was centrifuged at 8000×*g* for 15 min at 4 °C, and the supernatant was used directly (without pH adjustment or additional purification) for the subsequent decarboxylation reaction.

### 2.8 Whole-cell synthesis of 2-OH-PDA using decarboxylase SpLDC

The plasmid pETDuet-*SpLDC*, harboring the *SpLDC* gene, was transformed into *E. coli* BL21(DE3) for recombinant protein expression. Whole-cell biocatalysts were prepared by culturing the engineered strain in a 5-L bioreactor containing LB medium at 37 °C. Protein expression was induced at an OD_600_ of 0.4 by adding IPTG to a final concentration of 0.1 mM, followed by incubation at 18 °C for 14 h. Cells were harvested by centrifugation (8000×*g*, 15 min, 4 °C), washed with 50 mM phosphate buffer (pH 7.0), and re-suspended in the same buffer.

To systematically evaluate the effect of each factor on 2-OH-PDA synthesis, experiments were conducted by varying only the parameter under investigation while keeping other conditions constant. The effects of reaction temperature (25, 30, 37, 42, 47 °C), pH (5.0, 6.0, 7.0, 8.0, 9.0), and pyridoxal 5′-phosphate (PLP) concentration (0, 0.1, 0.25, 0.5, 1.0 mM) were investigated using crude fermentation broth containing 40.2 g/L 3-OH-lysine as substrate. All reactions were carried out with SpLDC whole-cell biocatalysts at a final OD_600_ of 10 (corresponding to 3.8 g DCW/L) at 200 rpm for 3 h, in triplicate.

### 2.9 Analytical methods

The concentration of glucose and l-lysine was determined using an SBA-10 immobilized enzyme biosensor (SIEMAN, Shenzhen, China). OD_600_ was determined by measuring the absorbance at 600 nm using a UV–visible spectrophotometer (AOELAB T6pc, Shanghai, China).

l-lysine, 3-OH-lysine, and 2-OH-PDA were analyzed by high-performance liquid chromatography (HPLC) (Shimadzu series) equipped with an Agilent HC-C18 reversed-phase column (5 μm, 25 cm × 4.6 mm) maintained at 50 °C. Prior to HPLC analysis, samples were derivatized using the Fmoc-Cl method: a 50 μL aliquot of sample solution (concentration ≤10 mM) was mixed with 250 μL of 100 mM borate buffer (pH 9.0), followed by addition of 300 μL of 10 mM Fmoc-Cl solution in acetone. The mixture was immediately vortexed for 10 s to ensure thorough homogenization and allowed to react for 30 min at ambient temperature in the dark. The reaction was quenched by adding 600 μL of 250 mM sodium borate solution containing 25% acetonitrile. The derivatized sample was centrifuged at 12,000 rpm for 2 min to remove precipitates, and the resulting supernatant was filtered through a 0.22 μm membrane prior to injection.

Chromatographic separation was achieved at a flow rate of 1.0 mL/min with an injection volume of 10 μL. The mobile phases consisted of mobile phase A: ultrapure water containing 0.1% trifluoroacetic acid (TFA); mobile phase B: acetonitrile supplemented with 0.1% TFA. The following gradient elution program was employed: 0–5 min, linear gradient from 50% to 90% B; 5–9 min, isocratic elution at 90% B; 9–12 min, linear gradient from 90% to 50% B; 12–13 min, column re-equilibration at 50% B. Detection was performed at a wavelength of 263 nm.

## 3 Results and discussion

### 3.1 Construction of 3-OH-lysine *de novo* synthetic strain

Previous production for 3-OH-lysine predominantly relied on exogenous l-lysine supplementation via *in vitro* enzymatic catalysis (e.g., KDO1, KDO2, or KDO5) or K3H-mediated whole-cell biocatalysis [2,14,19]. This imposes significant cost burdens, thereby limiting the industrial scalability and economic feasibility.

**Fig. 1:**
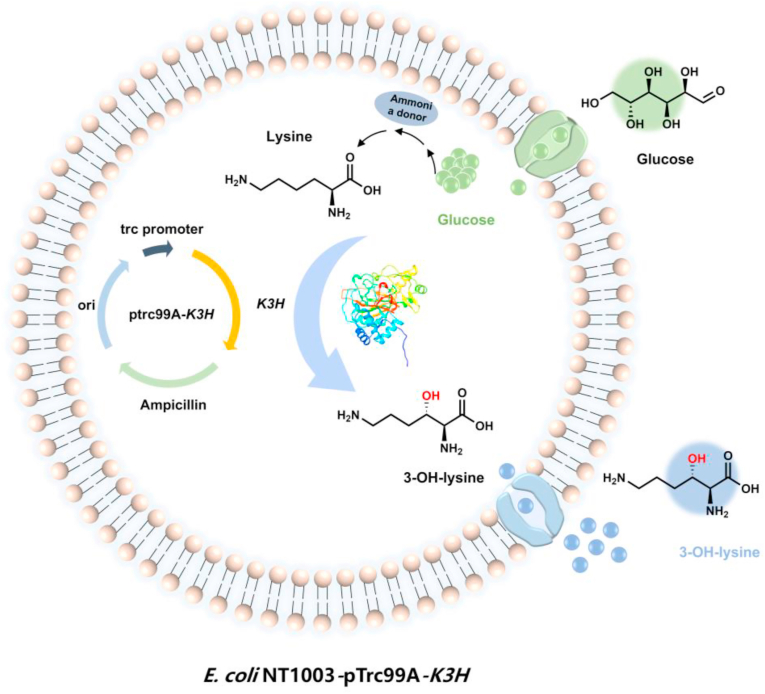
Schematic of the *de novo* 3-OH-lysine biosynthetic pathway in engineered *E. coli* NT1003-pTrc99A-*K3H.*

As shown in Fig. 1, the fermentation strain *E.* coli NT1003-pTrc99A-K3H was constructed for the *de novo* biosynthesis of 3-OH-lysine. Soluble expression of the hydroxylase K3H was confirmed by SDS-PAGE analysis (Fig. S1). The engineered strain produced 2.3 g/L of 3-OH-lysine within 24 h in shake-flask fermentation (Fig. S2), with the titer quantified by HPLC using a standard curve established with 3-OH-lysine purified in our previous study [20]. These results collectively confirm the successful construction of a functional 3-OH-lysine *de novo* biosynthetic strain.

### 3.2 Optimization of fermentation parameters for 3-OH-lysine production in shake flasks

**Fig. 2:**
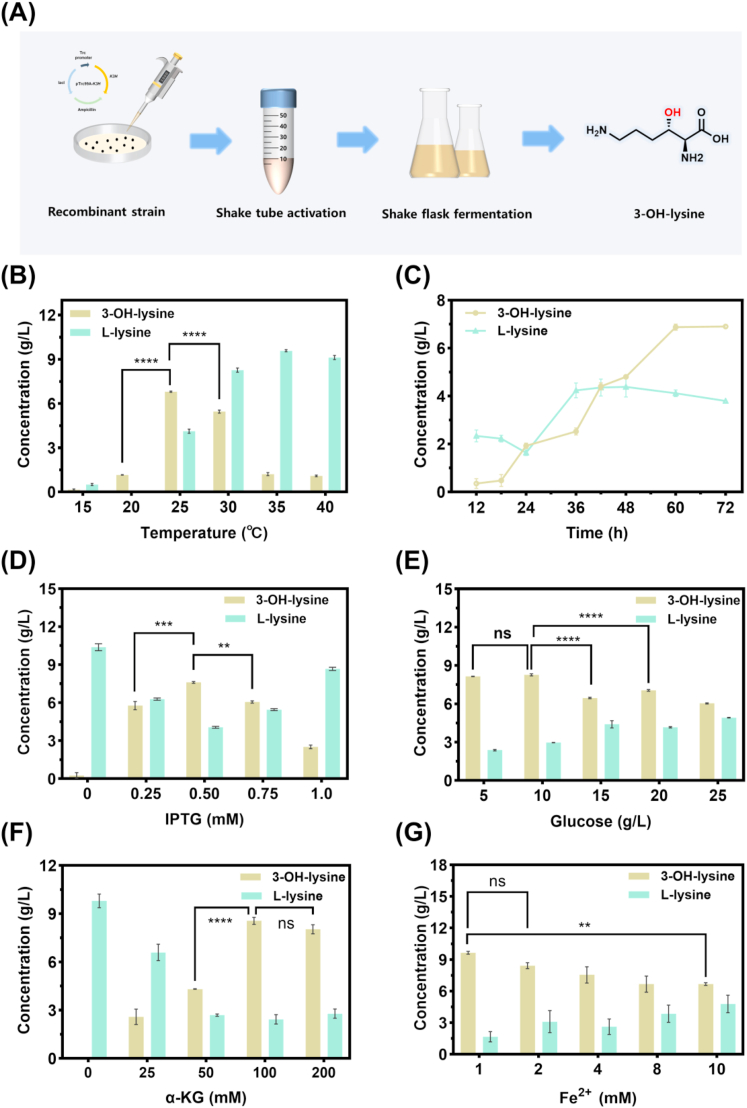
Effect of fermentation conditions on the production of 3-OH-lysine in shake flasks. Parameters were optimized sequentially: for each panel, only the indicated variable was varied, while all other fermentation conditions were held constant at their previously determined optimal values. (A) Schematic of the shake-flask fermentation process. (B) Effect of fermentation temperature. (C) Effect of fermentation time. (D) Effect of IPTG concentration. (E) Effect of initial glucose concentration. (F) Effect of α-ketoglutarate supplementation. (G) Effect of Fe^2+^ concentration. Data are presented as mean ± SD (n = 3). The statistical significance was determined by one-way ANOVA with Dunnett's multiple comparisons test. ns, P > 0.05; ∗∗P < 0.01; ∗∗∗P < 0.001; ∗∗∗∗P < 0.0001**.**

To improve 3-OH-lysine production, optimization of key parameters was conducted (Fig. 2A). As illustrated in Fig. 2B, the concentration of 3-OH-lysine increased as the induction temperature was raised from 15 °C to 25 °C, reaching a maximum of 6.78 g/L at 25 °C. Further increasing the temperature beyond 25 °C resulted in a decline in 3-OH-lysine production. This result suggests that K3H activity depends on proper protein folding and thermostability. Below 25 °C, slower protein synthesis rate limits the accumulation of active protein and relatively high temperatures increase protein misfolding and enzyme denaturation.

The effects of culture duration on 3-OH-lysine production was shown in Fig. 2C. The concentration of 3-OH-lysine increased from 12 h to 60 h, reaching a peak of 6.87 g/L. Between 60 h and 72 h, the accumulation continued at a reduced rate, likely due to precursor limitation or metabolic steady state. Nevertheless, 72 h was selected as the standard culture time for subsequent shake-flask experiments to maximize yield.

The effect of IPTG concentration was also evaluated (Fig. 2D). The titer of 3-OH-lysine increased with rising IPTG concentration from 0 to 0.5 mM, reaching a maximum of 7.61 g/L at 0.5 mM. Further increases in IPTG concentration beyond this point resulted in a decline in production. This phenomenon is likely because higher IPTG concentrations make proteins misfold into inclusion bodies and raise cellular toxicity, ultimately reducing 3-OH-lysine production [21].

As shown in Fig. 2E, the effect of glucose concentration on 3-OH-lysine production indicated that the optimal glucose concentration was 10 g/L, yielding a maximum titer of 8.27 g/L. A significant decrease in titer was observed above this concentration, this decline under glucose excess is consistent with catabolite repression, a well-documented phenomenon in *E. coli* where elevated glucose levels suppress the expression of non-preferred metabolic pathways through downregulation of cAMP [22].

Alterations in the α-KG concentration relative to 3-OH-lysine production are shown in Fig. 2F. The titer increased steadily with α-KG supplementation from 0 to 100 mM, reaching a maximum of 8.56 g/L at 100 mM. With further increases in concentration beyond 100 mM, the titer remained nearly constant.

Finally, Fe^2+^ concentration was optimized as it is essential for K3H activity. As presented in Fig. 2G, the titer of 3-OH-lysine reached the highest value of 9.65 g/L at 1.0 mM Fe^2+^, while a significant decrease occurred at 10 mM, likely due to metal ion toxicity. This observation is consistent with the report by Feng et al., which demonstrated that excessive metal ions could inhibit enzyme activity [23].

As a result, a 3-OH-lysine concentration of 9.65 g/L was obtained in shake-flask fermentation with induction temperature of 25 °C, cultivation time of 72 h, IPTG concentration of 0.5 mM, initial glucose concentration of 10 g/L, α-KG supplementation at 100 mM, and Fe^2+^ at 1 mM.

### 3.3 The optimization of the K3H gene expression strength

Fine-tuning the expression of rate-limiting enzymes represents a core strategy in metabolic engineering for enhancing product synthesis [[24], [25], [26]]. Thus, based on the optimal conditions in shake-flask fermentation (Section 3.2), the expression of the *K3H* gene was systematically optimized by engineering the RBS, promoter, and gene copy number.

**Fig. 3:**
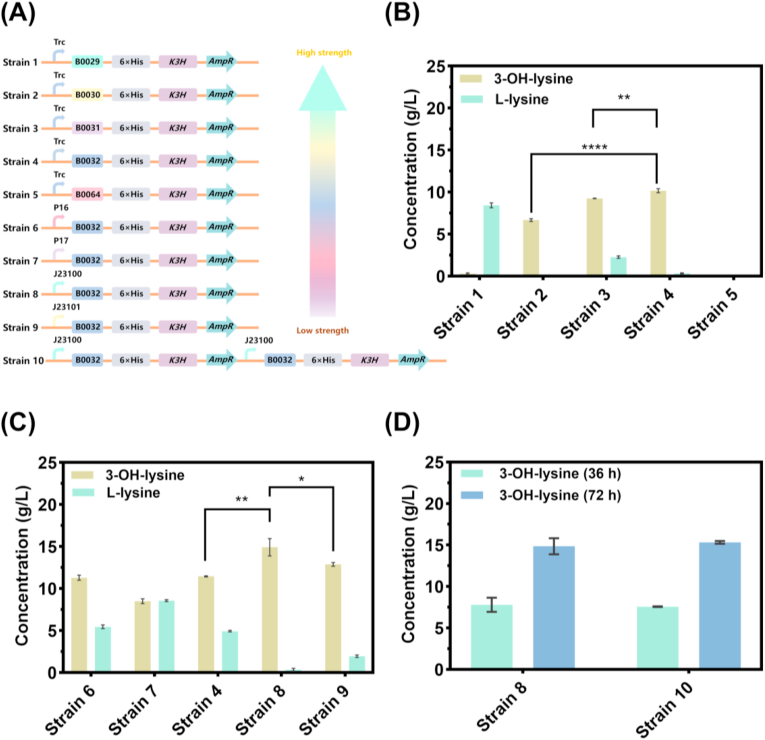
Optimization of *K3H* gene expression level. For each panel, only the indicated variable was varied, while all other genetic elements were held constant at their previously determined optimal configurations. (A) Genetic construction scheme for strains 1–10, with promoters and RBSs color-coded according to their relative strengths (see gradient arrows). (B) Effect of RBS strength on 3-OH-lysine production. (C) Effect of promoter strength on 3-OH-lysine production. (D) Effect of *K3H* gene copy number on 3-OH-lysine production. Data are presented as mean ± SD (n = 3). The statistical significance was determined by one-way ANOVA with Dunnett's multiple comparisons test. ∗P < 0.05; ∗∗P < 0.01; ∗∗∗∗P < 0.0001**.**

RBS engineering to regulate translation initiation is a widely used approach to balance enzyme expression and reduce metabolic burden [27,28]. In this study, five RBS variants with varying translation strength (B0029 > B0030 > B0032 > B0064 > B0031; Fig. S3) were selected and introduced into engineered strains (strains 1–5; Fig. 3A). As illustrated in Fig. 3B, strain 4 (carrying B0032) yielded the highest 3-OH-lysine titer of 10.1 g/L, representing a 9.7% increase over the native RBS control (strain 3, 9.2 g/L). In contrast, both stronger (strains 1–2) and weaker (strain 5) RBS variants resulted in reduced product accumulation. These findings suggest that an intermediate translation strength is most conducive to 3-OH-lysine synthesis, and that the relationship between translational strength and product titer is non-linear. This non-linear relationship reflects the trade-off between enzyme expression and folding. Strong RBS variants (e.g., B0029) overwhelm cellular folding capacity, causing K3H aggregation, while weak variants (e.g., B0031) limit expression below the flux requirement. B0032 provides an optimal balance to maximize active K3H, which provides the highest 3-OH-lysine production. These results were similar with previous studies [[29], [30], [31]].

Next, to further enhance transcription efficiency, five promoters with decreasing strengths (PJ23100 > PJ23101 > Trc > P16 > P17; Fig. S4) were evaluated in the background of strain 4, yielding strains 6–9 (Fig. 3A). As shown in Fig. 3C, results indicate that 3-OH-lysine yield increases with enhanced promoter strength. Strain 8 (PJ23100) achieved a maximum titer of 14.91 g/L, representing a 30% improvement over the original Trc promoter (strain 4). This implies that strong promoter-driven enhanced transcription elevates K3H mRNA levels and markedly boosts 3-OH-lysine synthesis, pointing to transcriptional capacity as a key bottleneck in the host strain.

To evaluate the effect of gene dosage on 3-OH-lysine synthesis. A dual-copy strain (Strain 10) was constructed by integrating an additional PJ23100-B0032-*K3H* expression cassette into the genome of strain 8 (Fig. 3A). As shown in Fig. 3D, no significant difference in 3-OH-lysine production was observed between strain 8 and strain 10 at either 36 h or 72 h. This suggests that increased gene dosage did not enhance enzyme expression or product formation, likely due to limitations in transcriptional/translational capacity or elevated metabolic stress. This result contrasts with certain systems where gene dosage positively correlates with protein expression levels [32], indicating that such a relationship is not universal.

Collectively, promoter strength had the most significant impact on 3-OH-lysine production, while moderate RBS strength was optimal and gene duplication provided no benefit. Therefore, strain 8, harboring the PJ23100 promoter, B0032 RBS, and single-copy *K3H* gene, was selected as the optimal strain for subsequent experiments.

### 3.4 Construction of an l-glutamate-driven α-KG supply system

**Fig. 4:**
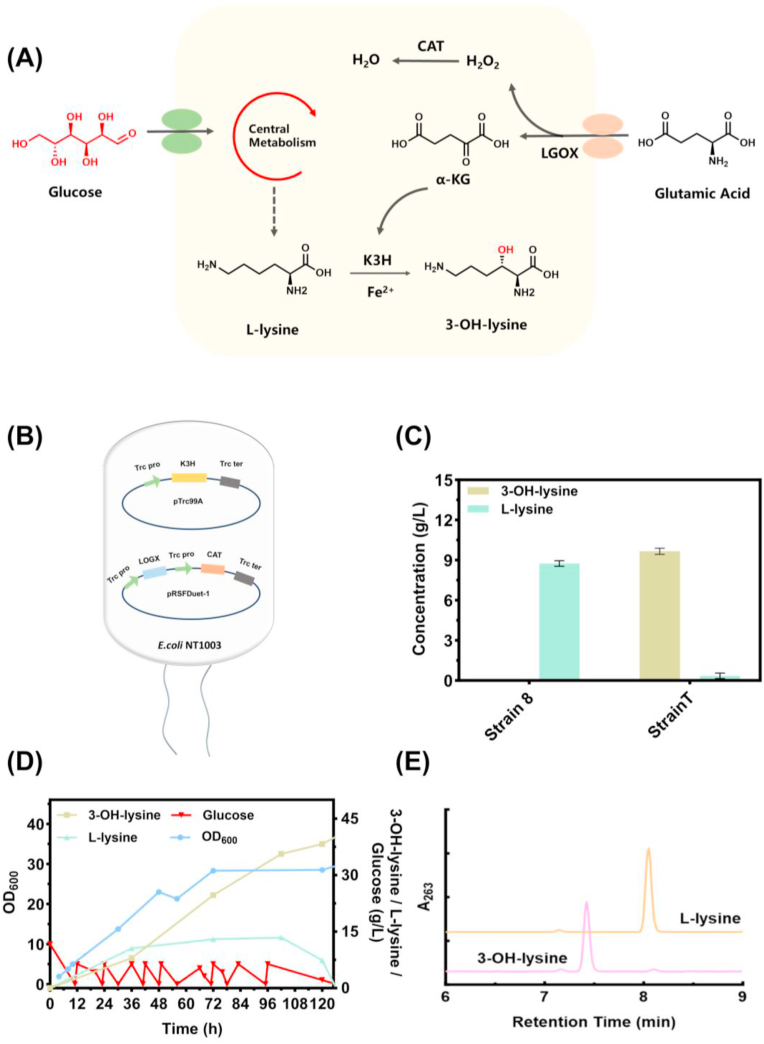
Cost-effective supply of α-KG via l-glutamate oxidation enables high-yield 3-OH-lysine production. (A) Schematic representation of the l-glutamate-driven α-KG supply pathway. (B) Construction of the dual-plasmid recombinant strain T. (C) 3-OH-lysine production by strain T vs. strain 8 control in the absence of exogenous α-KG. (D) Time-course profiles of key fermentation parameters in a 5-L bioreactor culture of the strain T. (E) HPLC analysis showing the consumption of l-lysine and production of 3-OH-lysine in the fermentation broth of strain T (pink), compared to an l-lysine standard (orange).

α-KG is an essential co-substrate for Fe^2+^/α-KG-dependent dioxygenases, such as K3H, which catalyzes the hydroxylation of l-lysine to 3-OH-lysine [2,33,34]. To circumvent the cost and scalability limitations associated with exogenous α-KG supplementation, an enzymatic regeneration system was constructed based on co-expression of LGOX and CAT (Fig. 4A). Successful co-expression of LGOX and CAT was verified by SDS-PAGE (Fig. S5). The design of engineered strain T, which incorporates this α-KG supply system along with the optimized K3H expression platform, is illustrated in Fig. 4B. This system substitutes costly α-KG (>50 USD/kg) with l-glutamate (∼2–3 USD/kg) and eliminates cytotoxic H_2_O_2_. Because α-KG (146.1 g/mol) and l-glutamate (147.1 g/mol) have nearly identical molecular weights and are interconverted in a 1:1 stoichiometric ratio, the molar cost of the α-KG equivalent decreases from ∼7.3 USD/mol to 0.3–0.4 USD/mol, a ∼20-fold reduction in raw material cost that highlights the economic advantage of this α-KG supply system.

Strain T and control strain 8 were cultured in shake flasks with l-glutamate alone for 72 h. Strain T produced 9.66 g/L of 3-OH-lysine, whereas control strain 8 produced l-lysine but no detectable 3-OH-lysine (Fig. 4C), confirming the essential role of the l-glutamate-driven α-KG synthesis system.

When cultured in a 5-L bioreactor, Strain T produced 40.2 g/L of 3-OH-lysine with negligible residual l-lysine (Fig. 4D and E). As shown in Table S3, the process consumed 209.74 g glucose and 110.35 g l-glutamate to yield 120.6 g of 3-OH-lysine, corresponding to yields of 0.575 g/g on glucose and 1.093 g/g on l-glutamate, with a volumetric productivity of 0.319 g/L/h. Additional performance metrics and comparisons with shake-flask fermentation are in Table S3. These results show that the fed-batch process achieves high titer, improves substrate efficiency, and enhances scalability compared to flask culture.

### 3.5 Whole-cell catalyzed synthesis of 2-OH-PDA

**Fig. 5:**
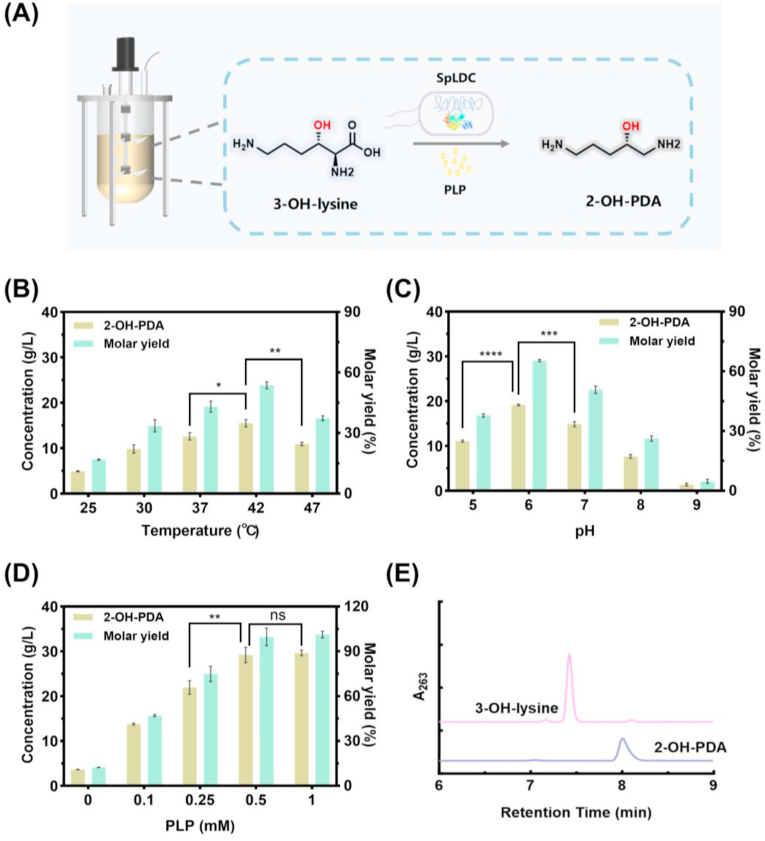
Optimization of SpLDC-catalyzed conversion of 3-OH-lysine to 2-OH-PDA. In each optimization experiment (B–D), only the indicated variable was varied, while all other catalytic conditions were held constant at their previously determined optimal values. (A) Schematic representation of the SpLDC-catalyzed decarboxylation of 3-OH-lysine to 2-OH-PDA. (B) Effect of reaction temperature on 2-OH-PDA yield. (C) Effect of pH on 2-OH-PDA production. (D) Impact of PLP supplementation on 2-OH-PDA synthesis. (E) Typical HPLC chromatograms for the 3-OH-lysine fermentation broth (pink) and the reaction mixture after SpLDC-catalyzed decarboxylation (light blue). Data are presented as mean ± SD (n = 3). The statistical significance was determined by one-way ANOVA with Dunnett's multiple comparisons test. ns, P > 0.05; ∗P < 0.05; ∗∗P < 0.01; ∗∗∗P < 0.001; ∗∗∗∗P < 0.0001**.**

A whole-cell biocatalytic system was developed using recombinant *E. coli* BL21(DE3) expressing the PLP-dependent decarboxylase SpLDC to enable 2-OH-PDA synthesis (Fig. 5A). To maximize enzymatic activity, the expression conditions of SpLDC were first optimized in shake flasks (Fig. S6). The optimal parameters were determined as induction at OD_600_ = 0.4 with 0.1 mM IPTG, followed by cultivation at 18 °C for 14 h, yielding whole-cell catalysts with the optimal activity. Based on this, key reaction parameters such as temperature, pH, and PLP concentration on 2-OH-PDA synthesis were systematically investigated in the whole-cell catalysis system. The 2-OH-PDA concentration was quantified using the standard curve established in our previous study [20].

As illustrated in Fig. 5B, 2–OH–PDA yield increased steadily from 25 °C to 42 °C, reaching a maximum of 15.5 g/L, beyond which a significant decline was observed due to apparent thermal denaturation of the enzyme.

The influence of reaction pH on 2-OH-PDA synthesis was shown in Fig. 5C. The highest titer of 19.2 g/L was achieved at pH 6.0, while activity decreased markedly under alkaline conditions and was completely abolished at pH 9.0.

Given the essential role of PLP as a cofactor for amino acid decarboxylases [35], its concentration was systematically optimized. As shown in Fig. 5D and E, the yield of 2-OH-PDA increased with PLP concentration in the range of 0–0.5 mM, reaching a maximum of 29.2 g/L at 0.5 mM, with a molar conversion yield of 99.7% achieved under this condition. Further increases in PLP concentration beyond 0.5 mM resulted in negligible changes in 2-OH-PDA production.

Notably, under the optimal conditions, a highly efficient conversion of 40.2 g/L of 3-OH-lysine to 29.2 g/L of 2-OH-PDA was achieved within 3 h with a remarkable 99.7% molar conversion rate and 0.726 g/g mass yield, suggesting near-complete carbon transfer from the intermediate to the product. The volumetric productivity of this step was 9.73 g/L/h, 5.8-fold higher than the 1.68 g/L/h reported by Li et al. for their decarboxylation step (80.5 g/L over 48 h). Although the reaction required 0.5 mM PLP as a cofactor, the industrial-scale cost is only ∼0.02–0.04 USD per liter of reaction mixture. Given the high value of 2-OH-PDA, which has potential applications in high-end industries such as pharmaceuticals and specialty chemicals, this cofactor cost is negligible. Moreover, ongoing research in enzyme engineering may lead to the development of more cost-effective enzyme systems that require lower cofactor amounts or can utilize alternative, cheaper cofactors.

Overall, this two-step process is a cost-effective, and scalable route for the biosynthesis of 2-OH-PDA and other multifunctional β-amino alcohols. To further enhance its industrial applicability, future research could focus on optimizing enzyme stability and activity through protein engineering and integrating this process with other bioprocessing steps to create a more integrated and sustainable production system.

## 4 Conclusion

In this work, we report a strategy for the first *de novo* biosynthetic pathway for 2-OH-PDA derived from glucose through a two-step fermentation–biocatalysis process. By integrating an l-lysine-overproducing *E. coli* chassis with K3H hydroxylase and a glutamate-driven α-KG supply module, we achieved 40.2 g/L 3-OH-lysine in a 5 L bioreactor, eliminating the need for exogenous l-lysine and costly α-KG. Subsequent whole-cell decarboxylation by SpLDC converted 3-OH-lysine into 2-OH-PDA with 99.7% molar yield (29.2 g/L). Although l-glutamate is supplied externally, it serves as a low-cost, scalable feedstock that reduces process complexity compared to α-KG. The modular strategy, which couples endogenous precursor overproduction, α-KG supply, and enzymatic cascade, offers a generalizable blueprint for biosynthesis of multifunctional amino alcohols.

## CRediT authorship contribution statement

**Zhijie Zheng:** Writing – original draft, Visualization, Methodology, Investigation, Formal analysis, Data curation, Conceptualization. **Zhongliang Chen:** Writing – review & editing, Methodology, Investigation, Formal analysis. **Tongqing Tang:** Investigation, Data curation. **Haoyu Lu:** Investigation, Data curation. **Qian Xu:** Investigation, Data curation. **Zixun Yang:** Investigation, Data curation. **Wei Liu:** Methodology, Investigation. **Chaoqiang Wu:** Methodology, Investigation. **Shewei Hu:** Writing – review & editing, Supervision. **Feifei Chen:** Writing – review & editing, Supervision. **Alei Zhang:** Writing – review & editing, Supervision, Project administration, Funding acquisition, Conceptualization. **Kequan Chen:** Writing – review & editing, Supervision, Project administration, Funding acquisition, Conceptualization.

## Availability of data and materials

All data generated or analyzed during this study are included in the manuscript and its Supplementary Information files. The recombinant strains and plasmids constructed in this work are available from the corresponding authors upon reasonable request.

## Declaration of competing interest

The authors declare that they have no known competing financial interests or personal relationships that could have appeared to influence the work reported in this paper.
