# Supplementary material for: De novo biosynthesis of 1,5-diamino-2-hydroxypentane from glucose via combination of fermentation and whole-cell catalysis
Source: Synth Syst Biotechnol. 2026 Feb 14;13:271–80. doi: 10.1016/j.synbio.2026.01.036 (PMC12925044; doi:10.1016/j.synbio.2026.01.036)
Supplement: Multimedia component 1 [file mmc1.docx]

**Supplementary Information**

***De novo* biosynthesis of 1,5-diamino-2-hydroxypentane from glucose via combination of fermentation and whole-cell catalysis**

Zhijie Zheng^†,‡^, Zhongliang Chen^‡^, Tongqing Tang^‡^, Haoyu Lu^‡^, Qian Xu^‡^, Zixun Yang^‡^, Wei Liu^‡^, Chaoqiang Wu^‡^, Shewei Hu^a^, Feifei Chen^†,‡^, Alei Zhang^*,†,‡^, Kequan Chen^*,†,‡^

^†^State Key Laboratory of Materials-Oriented Chemical Engineering, Nanjing Tech University, Nanjing 211816, China.

^‡^College of Biotechnology and Pharmaceutical Engineering, Nanjing Tech University, Nanjing 211816, China.

*Corresponding author: Alei Zhang, Kequan Chen

^a^Department of Biology and Food Engineering, Bozhou University, Bozhou 236800, China.

E-mail address: [zhangalei@njtech.edu.cn](mailto:zhangalei@njtech.edu.cn), [kqchen@njtech.edu.cn](mailto:kqchen@njtech.edu.cn)

**Contents Summary**

**Table S1**. Strains and plasmids used in this study.

**Table S2**. Primers used in this study.

**Table S3**. Comparion of 3-OH-lysine production performance between shake-flask and 5 L bioreactor cultures.

**Figure S1**. SDS-PAGE analysis of K3H expression in *E. coli* NT1003-pTrc99A-K3H.

Figure S2. Preliminary evaluation of 3-OH-lysine production in engineered *E. coli* NT1003-pTrc99A-*K3H* under shake-flask conditions.

Figure S3. Relative strengths of five RBS variants determined using a fluorescent reporter system.

Figure S4. Relative strengths of five promoters determined by RFP reporter assay.

Figure S5. Co-expression of LGOX and CAT in *E. coli* BL21(DE3)/pRSFDuet-*LGOX*-*CAT*.

Figure S6. Optimization of recombinant *SpLDC* expression in *E. coli* Bl21-pETDuet-*SpLDC.*

# Table S1. Strains and plasmids used in this study.

| **Strain/plaimsids** | **Characteristics** | **Source** |
| --- | --- | --- |
| Strains |  |  |
| *E. coli* DH5α | Cloning host | General Bio |
| *E. coli* BL21(DE3) | Expression host | General Bio |
| *E. coli* NT1003 | L-lysine-overproducing engineered strain | This lab |
| *Kocuria radiotolerans* | Wild type | General Bio |
| *Streptomyces viridosporus* | Wild type | General Bio |
| *Bacillus pumilus* | Wild type | General Bio |
| *Strain 1* | *E. coli* NT1003, (Trc) (B0029) *K3H* | This study |
| *Strain 2* | *E. coli* NT1003, (Trc) (B0030) *K3H* | This study |
| *Strain 3* | *E. coli* NT1003, (Trc) (B0031) *K3H* | This study |
| *Strain 4* | *E. coli* NT1003, (Trc) (B0032) *K3H* | This study |
| *Strain 5* | *E. coli* NT1003, (Trc) (B0064) *K3H* | This study |
| *Strain 6* | *E. coli* NT1003*,* (P16) (B0032) *K3H* | This study |
| *Strain 7* | *E. coli* NT1003*,* (P17) (B0032) *K3H* | This study |
| *Strain 8* | *E. coli* NT1003*,* (PJ23100) (B0032) *K3H* | This study |
| *Strain 9* | *E. coli* NT1003*,* (PJ23101) (B0032) *K3H* | This study |
| *Stain 10* | *E. coli* NT1003*,* (J23100) (B0032) *K3H*(J23100) (B0032) *K3H* | This study |
| *E. coli* DH5α-pRSFDuet-*K3H* | *E. coli* DH5α, *K3H* | This lab |
| *E. coli* Bl21-pRSFDuet-*K3H* | *E. coli* Bl21, *K3H* | This study |
| *E. coli* Bl21-pETDuet-*LGOX* | *E. coli* Bl21, *LGOX* | This study |
| *E. coli* Bl21-pACYC-*CAT* | *E. coli* Bl21, *CAT* | This study |
| *E. coli* DH5α-pRSFDuet-*LGOX*-*CAT* | *E. coli* DH5α, *LGOX* and *CAT* | This study |
| *E. coli* Bl21-pRSFDuet-Trc-*LGOX*-*CAT* | *E. coli* Bl21, (Trc) *LGOX*, (Trc) *CAT* | This study |
| *E. coli* NT1003-pRSFDuet-Trc-*LGOX*-*CAT* | *E. coli* NT1003, (Trc) *LGOX*, (Trc) *CAT* | This study |
| *E. coli* Bl21-pETDuet-*SpLDC* | *E. coli* Bl21, *SpLDC* | This lab |
| *Stain T* | *E. coli* NT1003, (PJ23100) (B0032) *K3H/* (Trc) *LGOX,* (Trc) *CAT* | This study |
| Plasmids |  |  |
| pRSFDuet-1 | Expression vector; with T7 promoter, Kan^R^ | General Bio |
| pETDuet-1 | Expression vector; with T7 promoter, Amp^R^ | This lab |
| pTrc99A | Expression vector; with T7 promoter, Amp^R^ | This lab |
| pACYCDuet-1 | Expression vector; with T7 promoter, Cap^R^ | This lab |
| Recombinant Plasmids |  |  |
| pRSFDuet-*K3H* | pRSFDuet-1, with *K3H* | General Bio |
| pTrc99A-*K3H* | pTrc99A (B0031), with *K3H* | This study |
| pTrc99A-B0029-*K3H* | pTrc99A (B0029), with *K3H* | This study |
| pTrc99A-B0030-*K3H* | pTrc99A (B0030), with *K3H* | This study |
| pTrc99A-B0031-*K3H* | pTrc99A (B0031), with *K3H* | This study |
| pTrc99A-B0032-*K3H* | pTrc99A (B0032), with *K3H* | This study |
| pTrc99A-B0064-*K3H* | pTrc99A (B0064), with *K3H* | This study |
| pTrc99A-P16-B0032-*K3H* | pTrc99A (P16) (B0032), with *K3H* | This study |
| pTrc99A-P17-B0032-*K3H* | pTrc99A (P16) (B0032), with *K3H* | This study |
| pTrc99A-PJ23100-B0032-*K3H* | pTrc99A (PJ23100) (B0032), with *K3H* | This study |
| pTrc99A-PJ23101-B0032-*K3H* | pTrc99A (PJ23101) (B0032), with *K3H* | This study |
| pTrc99A-PJ23101-B0032-*K3H-*PJ23101-B0032-*K3H* | pTrc99A (PJ23101) (B0032), with *K3H* and (PJ23101) (B0032), with *K3H* | This study |
| pETDuet-*LGOX* | pRSFDuet-1, with *LGOX* | This lab |
| pACYCDuet-*CAT* | pACYCDuet-1, with *CAT* | This lab |
| pRSFDuet-*LGOX*-*CAT* | pRSFDuet-1, with *LGOX* and *CAT* | This study |
| pRSFDuet-Trc-*LGOX-CAT* | pRSFDuet-1 (Trc), with *LGOX* and (Trc), with *CAT* | This study |
| pETDuet-*SpLDC* | pETDuet-1, with *SpLDC* | This study |

# Table S2. Primers used in this study.

| Primers | Sequence (5’→3’) |
| --- | --- |
| K3H-F | ACAATTTCACACAGGAAACAGACCATGGGCAGCAGCCATCAC |
| K3H-R | TAGAGGATCCCCGGGTACCGATTAGCTGAAGCTGGCCTGAAC |
| B0029-F | ATTCACACAGGAAACCCAGACCATGGGCAGCAGC |
| B0029-R | GGTTTCCTGTGTGAATTTGTTATCCGCTCACAATTCCAC |
| B0030-F | AATTAAAGAGGAGAAACAGACCATGGGCAGCAGC |
| B0030-R | TTTCTCCTCTTTAATTTTGTTATCCGCTCACAATTCCAC |
| B0032-F | ATCACACAGGAAAGCAGACCATGGGCAGCAGC |
| B0032-R | CTTTCCTGTGTGATTTGTTATCCGCTCACAATTCCAC |
| B0064-F | AAAAGAGGGGAAACAGACCATGGGCAGCAGC |
| B0064-F | TTTCCCCTCTTTTTTGTTATCCGCTCACAATTCCAC |
| B0032-P16-F | TTGACGATTAATCATCCGGCTCGTATGATGTGTGGAATTGTGAGC GGATAACAA |
| B0032-P16-R | CATCATACGAGCCGGATGATTAATCGTCAACAGCTCATTTCAGA ATATTTGCCAGAA |
| B0032-P17-F | TTTACAATTAATCATCCGGCTCGTACTATGTGTGGAATTGTGAGC GGATAACAA |
| B0032-P17-R | CATAGTACGAGCCGGATGATTAATTGTAAACAGCTCATTTCAGA  ATATTTGCCAGAA |
| B0032-PJ23100-F | TTGACGGCTAGCTCAGTCCTAGGTACAGTGTGTGGAATTGTGAG CGGATAACAA |
| B0032-PJ23100-R | CACTGTACCTAGGACTGAGCTAGCCGTCAACAGCTCATTTCAGA ATATTTGCCAGAA |
| B0032-PJ23101-F | TTTACAGCTAGCTCAGTCCTAGGTATTATGTGTGGAATTGTGAGC GGATAACAA |
| B0032-PJ23101-R | CATAATACCTAGGACTGAGCTAGCTGTAAACAGCTCATTTCAGA ATATTTGCCAGAA |
| CAT-F | ATGTGTGGAATTGTGAGCGGATAACAATTTTGTTTAACTTTAAG AAGGAGATATACCATGG |
| CAT-R | CCGCCAAAACAGCCAAGCTTGTTATTTCATACTACCCTGCAGAT AGCT |
| pTrc99A-F1 | CAAGCTTGGCTGTTTTGGCGG |
| pTrc99A-R1 | ACTCTAGAGGATCCCCGGGTA |
| LGOX-F1 | ATTTCACACAGGAAACAGACCATGAGCAGCCATCACCATCATCA C |
| LGOX-R1 | GTACCGAGCTCGAATTCCATGTTAGGCGGTATGAATTTCCAGGG C |
| LGOX-F2 | CATGGAATTCGAGCTCGGTAC |
| LOGX-R2 | CATGGTCTGTTTCCTGTGTGAAAT |
| Change the carrier-F | CCATTCGATGGTGTCCGGGAT |
| Change the carrier-R | TGCTTCTCAAATGCCTGAGGTTTCAGTTGCTTCGCAACGTTCAA ATCCGC |
| pRSFDuet-F | CTGAAACCTCAGGCATTTGAGAAGCA |
| PRSFDuet-R | ATCCCGGACACCATCGAATGG |

# Table S3. Comparion of 3-OH-lysine production performance between shake-flask and 5 L bioreactor cultures.

| **Parameter** | **Shake-flask (30 mL)** | **5-L fermentor ( 3 L working volume)** |
| --- | --- | --- |
| Culture time (h) | 72 | 120 |
| Glucose consumed (g) | 0.9 | 209.74 |
| L-Glutamate fed (g) | 0.662 | 110.35 |
| 3-OH-lysine (g) | 0.290 | 120.6 |
| Yield (g/g glucose) | 0.322 | 0.575 |
| Yield (g/g glutamate) | 0.437 | 1.093 |
| Yield (g/g glucose + glutamate) | 0.186 | 0.377 |
| Volumetric productivity (g/L/h) | 0.134 | 0.335 |

**
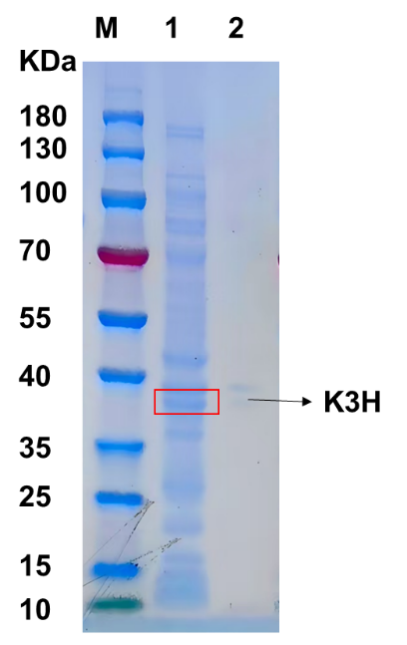
**

Figure S1. SDS-PAGE analysis of K3H expression in *E. coli* NT1003-pTrc99A-K3H. line M: Protein marker; line 1: K3H Soluble fraction (supernatant); line 2: K3H Insoluble fraction (pellet).

Figure S2. Preliminary evaluation of 3-OH-lysine production in engineered *E. coli* NT1003-pTrc99A-*K3H* under shake-flask conditions. (Control: *E. coli* NT1003; Experimental: *E. coli* NT1003-pTrc99A-*K3H*)

Figure S3. Relative strengths of five RBS variants determined using a fluorescent reporter system.

Figure S4. Relative strengths of five promoters determined by RFP reporter assay.


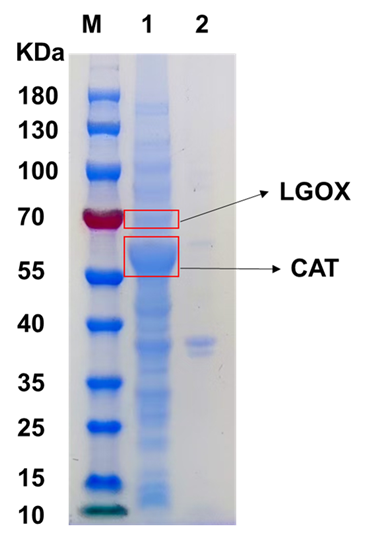


Figure S5. Co-expression of LGOX and CAT in *E. coli* BL21(DE3)/pRSFDuet-*LGOX*-*CAT*. lane M: Protein molecular weight marker; lane 1: Soluble fraction (supernatant); lane 2: Insoluble fraction (pellet).


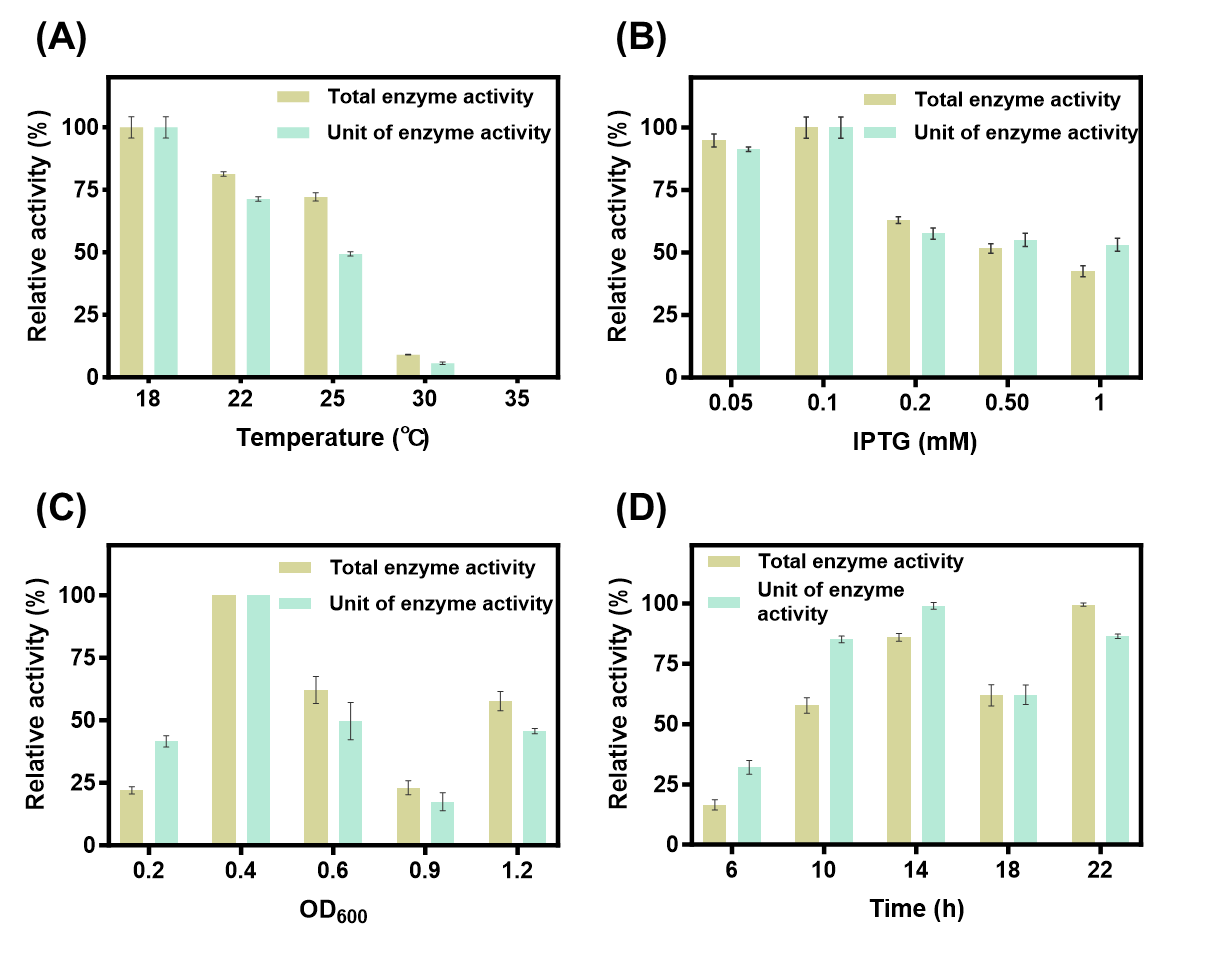


Figure S6. Optimization of recombinant *SpLDC* expression in *E. coli* Bl21-pETDuet-*SpLDC.* (A) Effect of induction temperature. (B) Effect of IPTG concentration. (C) Effect of cell density at induction. (D) Effect of induction duration. Results are presented as the mean of three biological replicates; error bars represent standard deviation.
